# When Pain Brings Gain: Soccer Players Behavior and Admissions Suggest Feigning Injury to Maintain a Favorable Scoreline

**DOI:** 10.3389/fpsyg.2016.00613

**Published:** 2016-04-28

**Authors:** Stuart W. G. Derbyshire, Ilana Angel, Richard Bushell

**Affiliations:** ^1^Department of Psychology, National University of SingaporeSingapore, Singapore; ^2^School of Psychology, University of BirminghamEdgbaston, UK

**Keywords:** pain, feigning, injury, malingering, football

## Abstract

The rules of soccer dictate that play, once halted, cannot continue if a player is injured. Players may take advantage of this rule by feigning injury to preserve beneficial match positions. Thirty Euro 2008 matches, 90 Premier League matches and 63 World Cup 2010 matches were reviewed for the timing and severity of injuries. The number of injuries was compared between teams that benefited from stopping the game and those that did not benefit. The number of low-level injuries, not resulting in substitution or subsequent problems, was directly compared for Benefit and Non-Benefit teams for each 15-min period following kick off. Statistical significance was assessed using appropriate non-parametric tests. In addition, seven current players and three managers were interviewed and were asked about feigning injury. Teams that benefited from game stoppages suffered significantly more minor injuries in the last 15 min of matches compared with those that did not benefit. Four of the players directly admitted feigning injury. When it is beneficial, soccer players can and do successfully feign injury to stop the game. Consequently it is possible that others might also successfully feign injury, pain or disease when motivated to do so.

## Introduction

Soccer is the most widely viewed and followed sport in the world. In 2009, the final of the European Champions League (Manchester United vs. Barcelona) attracted a total audience of 209 million viewers. The 2009 NFL Super Bowl (Pittsburgh Steelers vs. Arizona Cardinals), in contrast attracted a total audience of 162 million viewers (ViewerTrack, 2001)^1^. The 2010 FIFA World Cup Final (Spain vs. The Netherlands) attracted a total audience of 620 million people (Kantarsport, 2011)^2^ and each of the 2008 Euro Championship matches attracted a minimum audience of 155 million viewers (UEFA, 2008)^3^.

Accordingly, soccer generates enormous financial revenue and losing games can result in severe financial penalties. The failure of Manchester United to reach the knockout stages of the European Champions League in 2011, for example, was estimated to have cost the club more than £20 million in lost TV revenue and gate receipts (Dirs, 2011). The huge incentives to win games may encourage negative tactics such as feigning injury at the end of the game to run down the clock and maintain a winning position (Birdthistle, 2007). During World Cup 2006, “severe injury” delayed several games but the “severely injured” player requested a return to play just seconds after having left the pitch (Birdthistle, 2007).

Previous studies have examined the attitudes of youth soccer players using questionnaires and hypothetical scenarios describing likely in-play events including faking an injury (Kavussanu and Roberts, 2001; Ommundsen et al., 2003; Kavussanu, 2006, 2008). Although the reported frequency of negative behavior in youth soccer is low overall, studies do demonstrate that players report faking injury in order to gain an advantage. Moreover, negative behaviors increase when the motivational climate includes strong pressure to win, public recognition of ability and involvement in competitions (Ommundsen et al., 2003; Miller et al., 2005). All of these factors are likely to be engaged when professional soccer players are involved in high-pressure games.

Numerous previous studies have examined youth soccer but far fewer have considered the attitudes or behavior of professional soccer players (Hawkins and Fuller, 1999; Peterson et al., 2000; Birdthistle, 2007). The combination of pressures on professional soccer players, including pressure to win, public exposure and high-stakes competition, are unlikely to be precisely replicated in youth tournaments. These pressures lead to the prediction that professional players will change their behavior in a negative direction when a clear advantage emerges. One such situation includes the final minutes of a game where one team holds a favorable, but vulnerable, score. Under those circumstances, players who feign injury, slowing the game and potentially reducing the time for play, increase the potential for maintaining their team's favorable score.

Consequently, the current study examines the behavior of professional soccer players during three major tournaments—the English Premier League (EPL), the World Cup and the European Championships—and also includes interviews with professional players and managers from the EPL. The central aim is to assess whether players show evidence of feigning or exaggerating injury at the end of games where they have a favorable score. In the situation where one team is in front, or is drawing against a team expected to win, it is predicted that the frequency of injuries will increase for players from the team in the more favorable position. Similarly it is expected that players and managers might agree that such situations occur, and that feigning or exaggerating injury to run down the clock is an accepted strategy in professional soccer.

## Materials and methods

### Matches and participants

Thirty Euro 2008 soccer matches, 90 EPL matches and 63 World Cup 2010 matches were included. Thirty of the Premier League matches took place 20–30 December 2008 and the remaining 60 took place between August 2011–February 2012. During the last half of 2012, 15 current EPL players and seven managers were approached and asked if they would take part in an interview regarding match influences that may affect player behavior. Seven players and three managers provided written consent and were subsequently interviewed by one of the investigators (IA). All interview procedures were reviewed and approved by the University of Birmingham Central Ethics Committee.

### Procedure: match observation

All matches were recorded and individual matches viewed in a single sitting. Separate observers watched the Euro 2008/EPL 2008 matches and the World Cup competition (RB) and the EPL 2011/12 (IA) matches. Notes on the goals and injuries were recorded on a grid to indicate the time of the goal or injury, the team that scored or sustained the injury and the nature and severity of the injury.

Injuries that caused the game to be stopped were recorded and were classified as High, Medium or Low severity similar to previous research (Hawkins and Fuller, 1999). A High-severity injury was defined as any injury that caused the player to be substituted and subsequently miss at least 2 weeks of future training and matches. A Medium-severity injury was defined as any injury that caused the player to be substituted or caused the player to miss up to 2 weeks of training and subsequent matches. A Low-severity injury was defined as any injury that stopped play but did not result in the player being substituted or subsequently missing any training or matches. Low-severity injuries were expected to increase toward the end of a game for teams that could benefit from a delay in play and thus were entered into further analysis.

For analysis, the games were divided into 15-min periods: 0–15th min, 16–30th min, 31–45th min (including first-half stoppage time), 46–60th min, 61–75th min and 76–90th min (including second-half stoppage time). Mann Whitney *U*-tests were used throughout to assess differences in Low-severity injury rates for teams with and without a benefit for creating a delay in play. Mann Whitney was used because the data are non-parametric frequency data, which violate the assumptions for parametric analysis. For each game, low-level injuries were counted for each 15-min division and the number of injuries compared between benefit groups against the null-hypothesis of no difference. As six comparisons were performed, the critical *p*-value was set at *p* < 0.008 (*p* < 0.05 corrected for multiple comparisons). All comparisons were performed in SPSS using the Mann Whitney *U*-test legacy procedure, which is robust to violations of normalcy and equal distributions.

Benefit was primarily decided based on the current score (winning teams were always assigned as the Benefit team). In the event of the teams being level (as they always were at the start of any game) the Benefit team was assessed based on current league position or relative world rankings and on the relative benefit of a draw to each team and in-play factors such as player dismissals. For example, if Manchester United (a top EPL team) were playing Stoke City (a middle ranking EPL team) at Manchester then Stoke City will benefit more from a draw. Thus, Stoke City will begin the game as the Benefit team. Changes in the match situation in any 15 min period (for example, a goal or player dismissal) could cause the “Benefit team” to become the “Non-benefit team” and vice versa. In those situations, any future injuries were interpreted accordingly. For example, if Manchester United were leading 1-0 with 15 min left and suffered an injury in the 77th min that injury was recorded as a “Benefit” injury. If Stoke City then equalized in the 85th min and Stoke City suffered an injury in the 89th min, that injury was also recorded as a “Benefit” injury.

In all competitions, there were some matches in which there was no obvious benefit to either team in stopping the game in the final 15 min. Because the hypothesis pertains directly to the final 15 min of the match, these matches were designated as “Dead.” Dead matches included matches where the result would have no bearing on qualification due to previous results. This happened in the cup competitions where teams who had already qualified played teams that were already eliminated in the final group matches. Dead matches also included matches where one team was leading by at least three goals in the final 15 min. Although it is not impossible to overcome a three goal lead in the last 15 min of a match, such an outcome is highly unlikely and so the incentive for the Benefit team to delay play drops considerably for those games. Five matches from Euro 2008, five matches from the EPL 2008, 23 matches from World Cup 2010 and 16 matches from the EPL 2011-12 were judged to be Dead matches and were discarded for analysis.

### Procedure: interviews

Current EPL players and managers were first contacted through an agent to ask if they were willing to take part. Those who agreed were then directly contacted and a time arranged for the interview. All players and managers who were interviewed provided informed consent for their comments to be used in subsequent publication. Interviews took place face-to-face with the exception of one interview that took place over the phone. A further interview also required the presence of a translator. The experimenter recorded the interviews and later transcribed them at her own pace.

Interviewees were informed that the aim of the interview was to assess influences on player performance under real match circumstances. All interviewees were naïve to the research question. Accordingly, the following questions were asked of the seven players:

1. Do you feel that the referee is easily influenced by players?
2. Do you feel that the referee is easily influenced by home support/away jeering?
3. Do you feel a difference in play in yourself and your teammates if you play in a competition?
4. Do you feel a difference in play in yourself and your teammates at the beginning/end of the season?
5. Does the weather affect how you play?
6. Does the score-line affect how you play?
7. Does the time of game affect how you play?
8. What makes you respond more aggressively?
9. Do you have any strategies to “kill the clock” near the end of the first half/game? E.g., run the ball into the corner; pass the ball around more.
10. Do injuries hurt more/are worse at different points of the game? (E.g., as a result of fatigue)
11. Do you find that opposition players feign injury in order to win a free kick?
12. If so, does this usually depend on if they are in a winning position? Does this usually depend on the time of play?
13. Have you ever found yourself to play up an injury as worse that it is or go down easily in order to win a free kick or penalty? 14. If so, were you in a winning position? What time of the game was it?

And the following questions were asked of the three managers:

Do you feel that the referee is easily influenced by players?
Do you feel that the referee is easily influenced by home support/away jeering?
Do you feel that the referee is easily influenced by managers?
Do you instruct your players to play differently/motivate them in a different way in cup competitions?
Do you instruct your players to play differently/motivate them in a different way at the beginning and end of the season?
In what way does the score-line affect your tactics if the team you have out is playing at their best (but not necessarily winning)?
Does the time of game affect your tactics?
When would you expect your players to respond more aggressively?
Do you have any strategies to instruct your team to “kill the clock” near the end of the first half/game? E.g., run the ball into the corner; pass the ball around more.
Do you find that opposition players feign injury in order to win a free kick?
11. If so, does this usually depend on if they are in a winning position? Does this usually depend on the time of play?
Would you/have you ever instructed your players to play up an injury as worse than it is or to go down easily/stay on the ground for longer, in order to win a free kick or penalty?
If so, were you in a winning position? What time of the game was it?

After completion of the interview, all interviewees were informed of the hypothesis of the study and were reminded that they could withdraw any or all of their comments if they wished. No one requested withdrawal or modified their comments.

## Results

**Figure 1:**
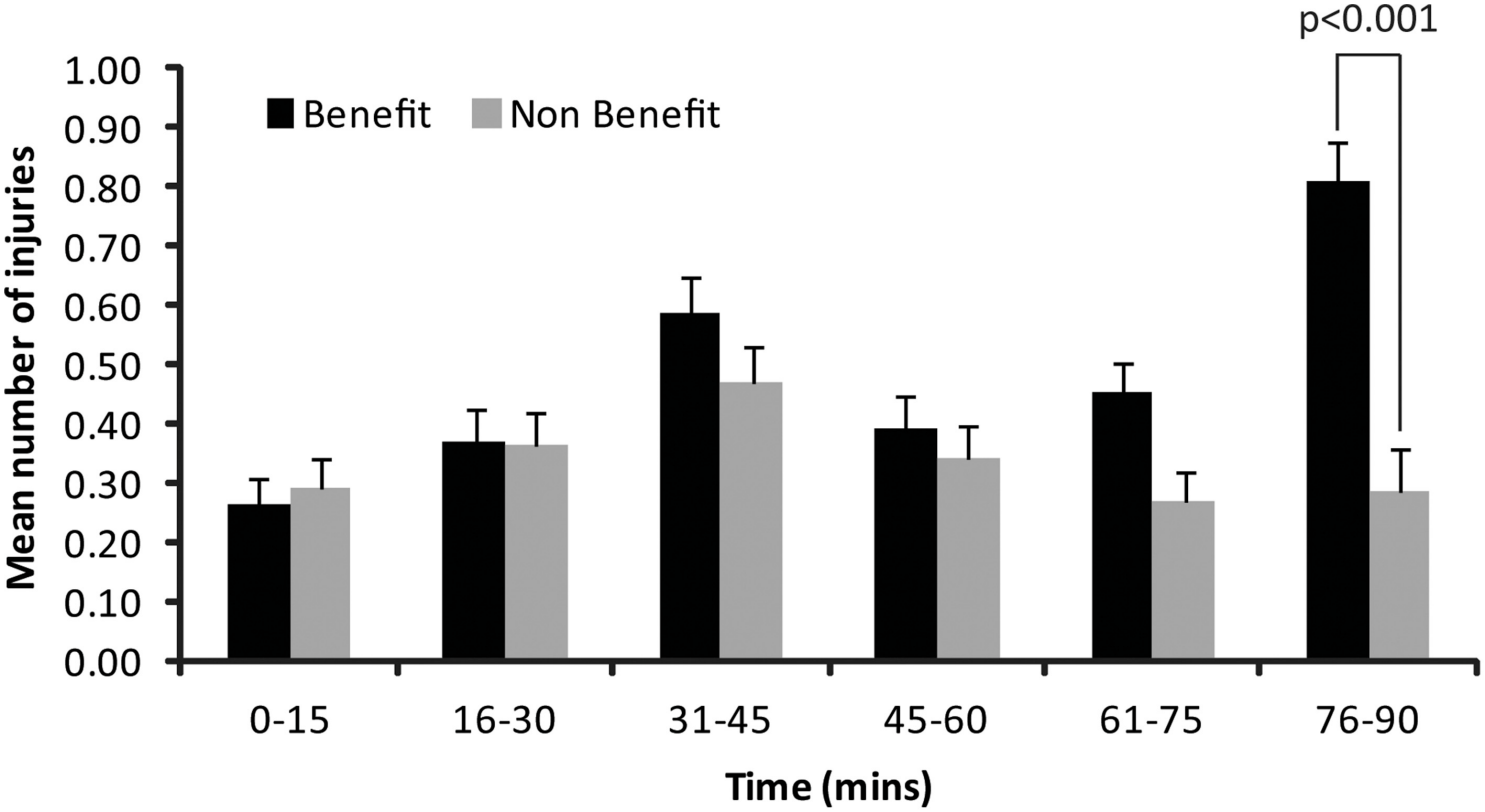
**Distribution of Low-severity injuries for teams that could benefit from slowing the game (Benefit) and teams that could not benefit (Non-Benefit). Error bars show standard errors**.

Figure 1 shows the mean injuries for the Benefit and Non-Benefit teams for each 15-min period. The differences were not significant in the first half (all p-thresholds are two tailed): 0–15 min (*U* = 8970, *p* = 0.70), 16–30 min (*U* = 9089, *p* = 0.90) and 31–45 min periods (*U* = 8377, *p* = 0.17). Differences were also not significant for the first period of the second half: 46–60 min period (*U* = 8976, *p* = 0.73) but the final two periods resulted in significantly more injuries for the “Benefit” team: 61–75 min (*U* = 7825, *p* = 0.01) and 76–9 min periods (*U* = 6518, *p* < 0.001, significant at *p* < 0.008). Cohen's *d* = 0.64 for the differences between Benefit and Non-Benefit teams in the final 15 min, which is an effect size in the upper range of medium.

The critical interview questions for the players were questions 11–14 and for the managers questions 10–13. These questions related to the central hypothesis concerning feigning injury and the answers are summarized in Table 1. Four of the seven players admitted actively “playing up” an injury to gain an advantage and six of the seven players suggested that opposition players feign injury. None of the three managers admitted to actively instructing their players to feign injury but all were somewhat ambivalent in their condemnation of such behavior. One described it as “clever,” another admitted that players did it, “to take the steam out of the game” and the third admitted they might tell a player to stay down, “and take a breather.”

**Table 1:** **Summarizes the answers to the final critical questions**.

**Question**	**Answers**
*Do you find that opposition players feign injury in order to win a free kick?* (Players) *Do you find that opposition players feign injury in order to win a free kick?* (Managers)	Yes. Every player. (P1) Players go down easily and win a free kick. (P2) Yer [yes], it happens in football…when I've trained with foreign teams, I have been asked why I didn't fake injury. (P3) A little bit…Players do do it. (P4) Yer [yes], 100% yer [yes]. (P5) There's a lot of speculation about diving and winning a cheap free kick but I don't think so. (P6) Yer [yes]. You get that all the time. (P7) Yes. When they are leading and away from home, they use every trick to waste time. (M1) Yer [yes]. More and more so now. (M2) It does happen. (M3)
*If so, does this usually depend on if they are in a winning position? Does this usually depend on the time of play?* (Players) *If so, does this usually depend on if they are in a winning position? Does this usually depend on the time of play?* (Managers)	It's whenever. (P2) Mainly when you're trying to get free kick or penalty. (P3) Yer [yes], if result is going for them…Last 10/15 min if losing, unlikely to feign an injury. (P4) Sometimes. (P5) Yes, definitely if they're winning and it's toward the end of the game to waste time. (P7) Doing it for a reason. To waste time…Good tactic to take steam out of the game. (M2) No. You see it from the first minute. (M3)
*Have you ever found yourself to play up an injury as worse that it is or go down easily in order to win a free kick or penalty?* (Players) *Would you/have you ever instructed your players to play up an injury as worse than it is or to go down easily/stay on the ground for longer, in order to win a free kick or penalty?* (Managers)	Yes, definitely. (P1) No. (P2) No, that's not me. (P3) I think sometimes you probably go down easier than you probably need do. (P4) Yer [yes]. (P5) No. Never and I will never do it. (P6) Yes. (P7) I don't instruct, but it's still clever. It depends on the importance of the game. (M1) No. Never instructed…Only time I did it was to take steam out of the game. (M2) I'm not saying experienced players in my team won't do it. Sometimes they go down and we need a breather and I might say, ok—you stay down there and take a breather. But I wouldn't suggest it before. (M3)
*If so, were you in a winning position? What time of the game was it?* (Players)	Yes, only at the end of a game. (P1) Sometimes. Just depends really. (P5) Yes, I was in a winning position. (P7)

*P, Players; M, Managers. There were no manager answers for the final question*.

## Discussion

This study used the naturally occurring, internal dynamics, of high-pressure soccer matches to predict an increase in the negative tactic of feigning or exaggerating injury toward the end of a game. The results of the study precisely support the prediction. During the first half of the observed matches, Low-severity injuries sustained by teams approaching a more favorable match outcome (Benefit teams) and by teams approaching a less favorable outcome (Non-Benefit teams) do not differ. During the second half of the observed matches, however, Low-severity injuries gradually increase for the Benefit team and gradually decrease for the Non-Benefit team. This difference in injury frequency reaches significance for the final 15-min game period. This pattern of results is exactly what would be predicted if a desire to slow the game down to gain an advantage from a delay in play drives Low-severity injuries. When asked directly, a majority of players agreed that they would feign injury to gain an advantage in a match, with several spontaneously associating the feigning of injury with slowing the game to maintain an advantageous position. Although no manager stated that they directly encouraged such behavior, with two categorically denying it, all admitted that they see the tactic used and understood why it is used. One manager specifically referred to it as “clever.”

Previous studies have also demonstrated that injuries in competitive soccer games tend to increase toward the end of each half (Hawkins and Fuller, 1999; Peterson et al., 2000). This may indicate that fatigue increases injuries late in a game. It is possible that fatigue varies with the Benefit and Non-Benefit team such that the Benefit team plays harder and is therefore more fatigued. While that is possible, we see it as unlikely. The critical games in this study were exactly those games where both sides had the potential for an improved outcome, and so both sides are expected to play at a high level of intensity. Nevertheless, fatigue was not directly addressed in the current study and is something that can be considered in the future.

Similarly, it is possible that some of the increase in injuries in the final part of the match is due to the extra time added at the end of the match. Soccer referees will add the time lost because of delays in play (due to injuries, substitutions and goals) to the end of each half and the average duration of stoppage-time added is 0.79 min in the first half and 2.93 min in the second half (Garicano et al., 2005). Consequently the final 15-min segment included in Figure 1 is always more than 15-min, which will inflate the number of injuries for that time period. Any inflation, however, should be the same for both Benefit and Non-Benefit teams and so the extra time cannot explain the discrepancy between the two teams.

Only one observer watched the matches, which creates the possibility of bias and denies us the opportunity to assess inter-rater reliability. Nevertheless, different observers watched different tournaments and the effects were consistent throughout. Observers were also not blind to the hypotheses or to which was the Benefit and Non-Benefit team. Future studies might consider using more than one observer and involving observers who are blind to the study aims.

We infer that the discrepancy between Benefit and Non-Benefit teams in the final 15 min is due, at least in part, to players feigning pain and injury when there is a benefit to slowing the game. By going to ground, a player encourages the referee to give a foul or stop the game while the player receives treatment. The precise effectiveness of the tactic, as well as its frequency, might be assessed in future studies by measuring the time of delay or the number of fouls awarded the Benefit team. Future studies might also consider other time-wasting tactics such as the goalkeeper deliberately holding the ball, players leaving the field slowly during substitution, and delaying the start of play when taking corners, free kicks and throw ins.

Typically, the Benefit team player would go to ground following contact with another player, including grimacing, holding the injured area and signaling for assistance from the bench. It is possible, therefore, that the player received a genuine knock, which was then exaggerated. Thus, the player might be accused of feigning the severity of the injury rather than the entire injury. Future studies might further address that distinction. Regardless of whether the player receives a knock or not, however, becoming more readily immobile requires an explanation. One explanation is that immobilization brings a benefit to the team.

It is known that people can and do change their behavior according to the gains brought about by the changes. As others have noted, financial benefit increases the likelihood of patients being referred for tests, screening procedures or operations. Cesarean section rates are higher when private practitioners, paid per operation, provide the care for pregnant women (Levitt and Dubner, 2005; Smith, 2006). Soccer players in a winning team, or holding a draw against expectation, have an incentive to stop play and maintain their position as the game goes forward. Here we provide compelling evidence, both behavioral and self-report, that one way this is achieved is through the successful feigning of injury. We raise the question of whether others might also successfully feign injury, pain or disease when motivated to do so (Halligan et al., 2003).

Prior studies have noted that sport can be used as a vehicle for character development and to develop prosocial behavior, especially amongst the young (Kavussanu, 2006). The time-wasting behavior described here could be described as unsportsmanlike and immoral, and something not to be emulated by young people involved in sport. While we recognize the potential merit in that position, we hesitate to call feigning or exaggerating injury anything more than negative. Professional soccer is not youth soccer. Whereas youth soccer may involve character building, professional soccer is not, on the whole, concerned with developing the moral character of the players. Professional soccer is a highly lucrative form of entertainment where the rewards for winning, and the punishments for losing, can be staggering. Part of the guiding morality of a professional soccer club includes remaining financially viable, and winning ugly games supports financial viability. Another part of the guiding morality of a professional soccer club includes keeping the fans happy. As fans of the game, we vent frustration when a player from an opposing side engages in an obvious tactic to slow the game and waste time. But we also recognize when our own players do this that it is a beneficial tactic, which, as demonstrated here, is recognized by players and managers as “clever.” Fans are happy when their team wins, even if the win is ugly.

## Conclusions

Professional soccer players in a winning or beneficial draw position increasingly show signs of mild injury as the game draws to the end. This behavior, and their freely given comments, suggest a deliberate strategy to feign injury for gain.

## Author contributions

Conceived and designed the experiments: SD, IA, RB. Performed the experiments: IA, RB. Analyzed the data: IA, SD. Wrote the paper: SD, IA, RB

### Conflict of interest statement

The authors declare that the research was conducted in the absence of any commercial or financial relationships that could be construed as a potential conflict of interest.

## Footnotes

^1^ViewerTrack (2010). http://www.influencia.net/data/document/viewertrack-2010.pdf [accessed *Nov* 26, 2015].

^2^KantarSport (2011). http://www.fifa.com/mm/document/affederation/tv/01/47/32/73/2010fifaworldcupsouthafricatvaudiencereport.pdf [accessed Nov 26, 2015].

^3^UEFA Euro 2008 Review (2008). http://www.uefa.com/MultimediaFiles/Download/MediaRelease/Competitions/MediaServices/73/54/28/735428_DOWNLOAD.pdf [accessed Nov 26, 2015].
